# Epidemiological Spectrum of Bovine Tick-Borne Pathogens in Northeast Brazil: Comparative Analysis Across a Tropical Humid and Two Semi-Arid Regions

**DOI:** 10.3390/pathogens15010015

**Published:** 2025-12-22

**Authors:** Felipe Boniedj Ventura Alvares, Jordania Oliveira Silva, Basilio Felizardo Lima Neto, Geraldo Moreira Silva Filho, Samira Pereira Batista, Marcelo Bahia Labruna, Thais Ferreira Feitosa, Vinícius Longo Ribeiro Vilela

**Affiliations:** 1 Postgraduate Program in Science and Animal Health, Federal University of Campina Grande—UFCG, Patos 58055-018, PB, Brazil; felprathalos@gmail.com (F.B.V.A.); basilio.felizardo@academico.ifpb.edu.br (B.F.L.N.); geraldosfilho4@gmail.com (G.M.S.F.); sammirabatista@hotmail.com (S.P.B.); thais.feitosa@ifpb.edu.br (T.F.F.); 2 Department of Veterinary Medicine, Federal Institute of Paraíba—IFPB, Sousa 58805-345, PB, Brazil; jordaniaoliveira6091@gmail.com; 3 Department of Preventive Veterinary Medicine and Animal Health, Faculty of Veterinary Medicine, University of São Paulo—USP, São Paulo 05508-270, SP, Brazil; labruna@usp.br

**Keywords:** anaplasmosis, babesiosis, cattle tick fever, risk factors

## Abstract

Cattle tick fever (CTF), caused by *Anaplasma marginale*, *Babesia bovis* and *Babesia bigemina*, remains a sanitary and economic challenge for cattle farming in Brazil. Thus, this study evaluated the prevalence, regional distribution, co-infection patterns, and risk factors associated with CTF causative agents in cattle the semi-arid region of Paraíba, the semi-arid region of Ceará, and the Tropical Humid region of Paraíba, Northeast Brazil. Blood samples were collected from 336 cattle, from 60 farms, and analyzed by means of conventional PCR and nested-PCR, while epidemiological data were obtained through questionnaires applied to producers. The overall infection prevalence by at least one pathogen was 82.7% (278/336), with higher rates in the tropical humid region of Paraíba at 94.8% (109/115), followed by the semi-arid region of Ceará, with 88.1% (89/101) and the semi-arid region of Paraíba with 66.6% (80/120). Co-infections were frequent, especially the association between *A. marginale* and *B. bigemina*, detected in 23.2% (78/336) of the animals, while triple infections occurred in 15.8% (53/336) and were most frequent in the semi-arid region of Ceará at 21.8% (22/101). The semi-arid region of Paraíba had the fewest entirely positive properties (7/20) and the highest number of entirely negative properties (2/20). The tropical humid region of Paraíba had the highest number of entirely positive properties (17/21), with no properties entirely free of CTF agents. Multivariate analysis identified the presence of horn fly (OR = 7.23; CI 3.05–18.86; 95% CI), needle reuse (OR = 5.8; CI: 2.62–13.90; 95% CI), animal purchase and introduction without quarantine (OR = 5.4; CI: 2.17–14.93; 95% CI), and pasture sharing (OR = 3.21; CI: 1.08–11.25; 95% CI) as risk factors, while beef herds showed lower susceptibility (OR = 0.28; CI: 0.15–0.52; 95% CI). These findings demonstrate that infections by CTF causative agents are endemic but exhibit region-specific epidemiological patterns, reflecting the combined effects of climate and management practices, and localized transmission foci that may be intensified by commercial cattle movement.

## 1. Introduction

Cattle farming is one of the main agricultural activities in Brazil, playing a fundamental role in generating income in rural areas and contributing significantly to the national economy [1]. Despite its relevance, the sector faces serious health challenges that directly affect herd productivity, especially in regions with adverse environmental conditions, such as Northeast Brazil [2]. Among the diseases with the greatest impact in this scenario is cattle tick fever (CTF), a parasitic complex considered one of the main causes of morbidity and mortality in cattle, especially in extensive farming systems [2,3].

Cattle tick fever is caused by three hemoparasites that affect bovine erythrocytes: *Babesia bovis* and *Babesia bigemina* (protozoa of the phylum Apicomplexa) and *Anaplasma marginale* (bacterium of the order *Rickettsiales*) [4,5]. These agents can occur alone or concomitantly, resulting in clinical manifestations that include fever, apathy, hemolytic anemia, jaundice, hemoglobinuria, decreased milk production, and, in severe cases, sudden death [6,7]. In addition to clinical signs, complications such as delayed weight gain and prolonged rearing period are frequent, which directly compromise the zootechnical performance of herds [8].

The economic losses resulting from CTF are significant, encompassing direct losses, such as animal mortality and decreased productivity, and indirect losses, such as expenses from medication, diagnostic tests, and health management [6,8]. Globally, tick-borne diseases, including CTF, are estimated to generate billions of dollars in annual losses [9]. In Brazil, ticks and blood parasites alone are associated with losses exceeding USD 2 billion per year [10].

In Northeast Brazil, varying climatic conditions strongly influence the epidemiology of parasitic diseases. In the coastal strip, characterized by a humid tropical climate, a high prevalence of blood parasites has been observed in beef cattle, particularly *A. marginale* [7]. Inland areas, however, have a semi-arid climate, with high temperatures, low humidity, and irregular rainfall, factors that also modulate the dynamics of CTF vectors [2]. In this context, as the most frequent infectious disease in cattle [11], and in a retrospective analysis of 24 outbreaks in the semi-arid region of Paraíba, more infections by *A. marginale* than by *Babesia* spp. were observed [12].

In addition to climatic factors, which influence the dynamics of CTF in cattle populations, infections by the etiological agents of this disease exhibit distinct epidemiological behaviors. While *Babesia* spp. infections are directly associated with the presence and dynamics of its biological vector, *Rhipicephalus microplus*, *A. marginale* infections can be transmitted more broadly, including mechanically by hematophagous insects such as horn flies (*Haematobia irritans*) and stable flies (*Stomoxys calcitrans*), as well as iatrogenically during veterinary procedures [13,14]. From an epidemiological perspective, *B. bovis* and *B. bigemina* have distinct pathogenicity and outbreak-causing behaviors, with *B. bovis* generally associated with more severe, rapidly evolving conditions [15].

Infections by CTF agents can occur alone or in combination, resulting in simple or mixed cases in herds [14]. This distinction has practical implications, as the recommended treatment differs depending on the agent involved, with specific drugs for *Babesia* spp., *A. marginale*, or combined action [16]. Babesiosis is typically managed with imidocarb dipropionate, whereas anaplasmosis is treated with tetracycline-based antimicrobials. However, none of these drugs consistently eliminates persistent infections, and treatment success depends heavily on early diagnosis and supportive clinical management [7,16].

Molecular epidemiology has become essential for the detection and characterization of bovine tick-borne pathogens, primarily because traditional diagnostic approaches such as blood smear evaluation exhibit limited sensitivity in chronically infected or carrier animals [6,8,11]. PCR-based assays, including nested PCR protocols targeting species-specific genomic regions, allow the identification of *A. marginale*, *B. bovis*, and *B. bigemina* even at low parasitemia levels, thereby improving the accuracy of prevalence estimates and revealing coinfection patterns that would otherwise remain undetected [8,15]. Despite this, there are no epidemiological studies that systematize the assessment at the herd level by using PCR-based assays in the Northeast region of Brazil, classifying farms according to the absence, singular, or combined presence of agents, considering the diversity of infections that can occur simultaneously.

In Northeastern Brazil, different climate regimes, such as those found in the semi-arid region and the humid tropical coastal strip, directly influence the dynamics of CTF [2]. Despite this, studies that systematically compare these regions to demonstrate how environmental conditions modulate the occurrence of CTF are still scarce.

In addition to climatic differences, risk factors related to herd management and composition also modulate the epidemiology of CTF. Characteristics such as age, breed, and biosecurity practices influence animal susceptibility and the persistence of agents in herds, potentially resulting in different infection scenarios within farms [4,17].

Given this context, the present study aimed to investigate the prevalence and risk factors of CTF agents in different regions of Northeastern Brazil, contrasting areas with semi-arid and tropical humid climates. Furthermore, we sought to characterize the epidemiological diversity at the farm level, considering the extent of infections and the occurrence of different combinations of agents, in order to provide a more comprehensive view of the regional dynamics of CTF.

## 2. Materials and Methods

### 2.1. Study Area

The study was conducted in the Northeast region of Brazil, in the semi-arid and tropical humid climate regions, in the states of Paraíba (PB) and Ceará (CE). The semi-arid region, located in western Paraíba and southern Ceará, is characterized by long periods of drought, in which most of the rainfall is concentrated between February and May and annual rainfall is concentrated between 400 and 800 mm [18], with extremes ranging from 244 to 1488 mm [19]. Average annual temperatures fall between 26 °C to 30 °C, with a low annual temperature range, and average annual relative humidity is 60%, ranging from 41.6% in July to 85.9% in February [19].

The tropical humid region of Paraíba, on the other hand, located mainly on the coastal strip in the east of the state, has significantly higher annual precipitation than the semi-arid region, ranging from 1200 mm to over 1800 mm [20,21]. Furthermore, in this region, relative humidity remains high throughout the year, often above 75% during the rainy season [21]. In the tropical humid region of Paraíba, average annual temperatures remain high throughout the year, ranging from 24 °C to 28 °C, with small seasonal variation. The warmest months usually occur between December and March, while the lowest averages are recorded between June and August [20].

### 2.2. Experimental Sampling

The study was carried out in the semi-arid region of the states of Paraíba and Ceará and in the tropical humid region of Paraíba. Initially, the number of animals to be collected per region was selected through simple random sampling, according to Thrusfield [22]:

$$n=\frac{z^{2}\;\cdot \;P\;(1-P)}{d^{2}}$$

where:

n: Number of samples selected

z: 1.96 (95% confidence level)

P: Expected prevalence (50%)

d: Standard error (10%)

Adjusted to the local population using the formula:

$$n_{ajus}=\frac{N*n}{N+n}$$

n_ajus_: Final number of selected samples

n: Number of selected samples

N: Number of existing samples

From this calculation, it was determined that it would be necessary to collect samples from at least 96 animals in each region. The second stage was to determine the number of animals to be collected per farm based on the detection of infections in the herd, according to Thrusfield [22]:

$$n_{ani}=\left\{1-\left(1-p^{\frac{1}{d}}\right)\right\}\cdot \left\{N-\frac{d}{2}\right\}+1$$

where:

n_ani_: Required sample size

N: Farm population size

d: Number of affected animals in the population (20%) [11]

*p*: Probability of finding at least one case in the sample (95%)

**Figure 1:**
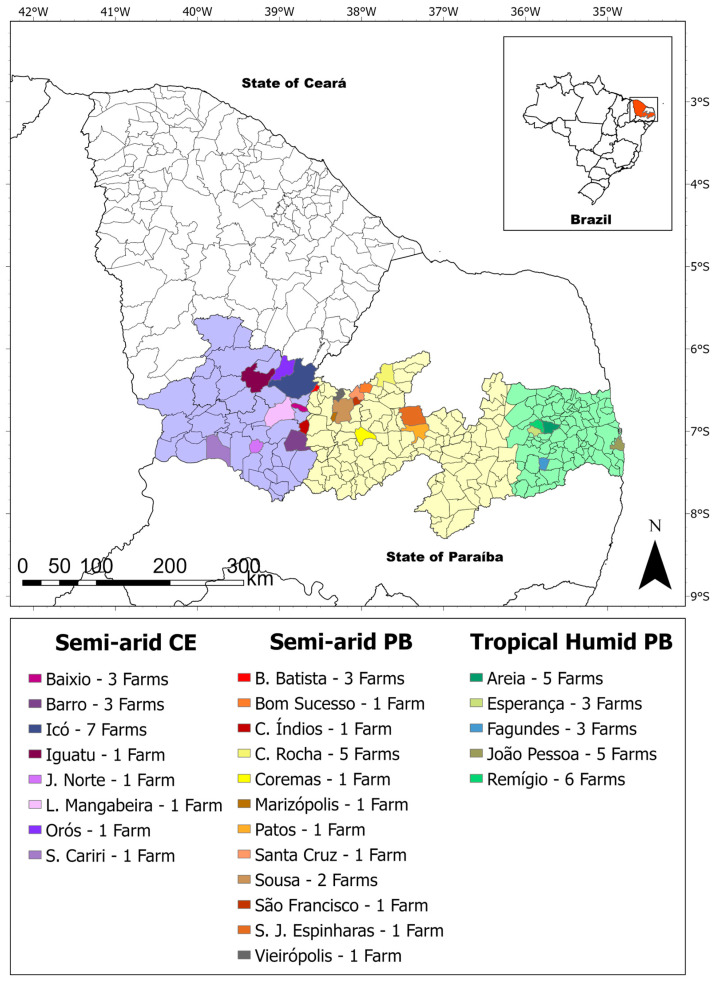
Geographic distribution of sampled cattle farms in the semi-arid and tropical humid regions of Northeast Brazil.

Thus, farms with up to 20 cattle had samples collected from 5 animals; farms with more than 20 cattle had 6 samples collected. Thus, 120 samples were collected from 20 farms in the semi-arid region of Paraíba, 101 samples from 18 farms in the semi-arid region of Ceará, and 115 samples from 22 farms in the tropical humid region of Paraíba, totaling 336 samples from 60 properties (Figure 1). The samples were collected from ≥12 months old animals.

### 2.3. Blood Sample Processing

Samples were collected via jugular or caudal venipuncture in 5 mL tubes containing EDTA and stored at 2–8 °C until laboratory processing. After centrifugation for 15 min at 1500× *g*, the plasma was removed and 400 µL aliquots of cell concentrate were transferred to 1.5 mL microtubes, labeled, and frozen at −20 °C for up to seven days until DNA extraction from the samples.

DNA from bovine blood samples was extracted using a commercial extraction kit (PureLink genomic DNA mini kit^®^, Invitrogen, Thermo Fisher Scientific, Waltham, MA, USA), following the manufacturer’s instructions.

### 2.4. Molecular Diagnosis

PCR reactions were performed using the primers described by Casa et al. [23] for *A. marginale* and their adaptation of Figueroa et al. [24] for *B. bovis* and *B. bigemina*. For the diagnosis of *A. marginale*, *B. bovis,* and *B. bigemina*, the primers used and thermocycler conditions are described in Table 1. The thermocycler conditions were adapted from Casa et al. [23]. For a final volume of 20 μL, 2 μL of DNA (the DNA concentration ranged from 60 to 107 ng/μL) were added to 3.15 μL of Platinum™ TaqDNA Polymerase mix (Invitrogen, Carlsbad, CA, USA), 12.85 μL of ultrapure water (dH_2_O), and 1.0 μL of each primer (10 μM).

### 2.5. Epidemiological Questionnaire

To determine the prevalence and distribution of CTF on the properties, each producer answered an epidemiological questionnaire about the following items: cattle population, animal breed, production system (intensive, extensive, or semi-intensive), main aptitude (dairy or beef cattle) and grazing area for the animals. They were also asked about what they considered to be the main health problem on the property, whether there had been any cases of CTF on the property, whether the needle was reused after applications, whether there was the presence of horseflies (*Tabanidae*), stable flies (*Stomoxys calcitrans*), and horn flies (*Haematobia irritans*), whether pasture was shared with other cattle herds, whether there was contact between cattle and other herds, whether there was rotation of bulls between farms for reproductive purposes, and how often animals were acquired for the herd.

### 2.6. Statistical Analysis

The prevalence and risk factors associated with *A. marginale*, *B. bovis*, and *B. bigemina* infections were determined by comparing PCR results with epidemiological questionnaire data in two stages: univariate and multivariate analysis. In the univariate analysis, each independent variable was cross-referenced with the dependent variable (positivity), and those with a *p*-value ≤ 0.05 determined using the chi-square test were selected for multivariate analysis using multiple logistic regression [25]. The significance level adopted in the multivariate analysis was 5%. The results were analyzed using GraphPad Prism 9.5.0.

## 3. Results

An overall prevalence of 79.7% (278/336) for at least one of the three agents was observed, with rates of 66.4% (223/336) for *A. marginale*, 50.5% (170/336) for *B. bigemina,* and 33% (111/336) for *B. bovis*. Agents of the CTF complex were detected in most cattle, with high prevalence in the tropical humid region of Paraíba (94.8%; 109/115), followed by the semi-arid region of Ceará (88.1%; 89/101), and the semi-arid region of Paraíba (66.6%; 80/120) (Table 2).

**Figure 2:**
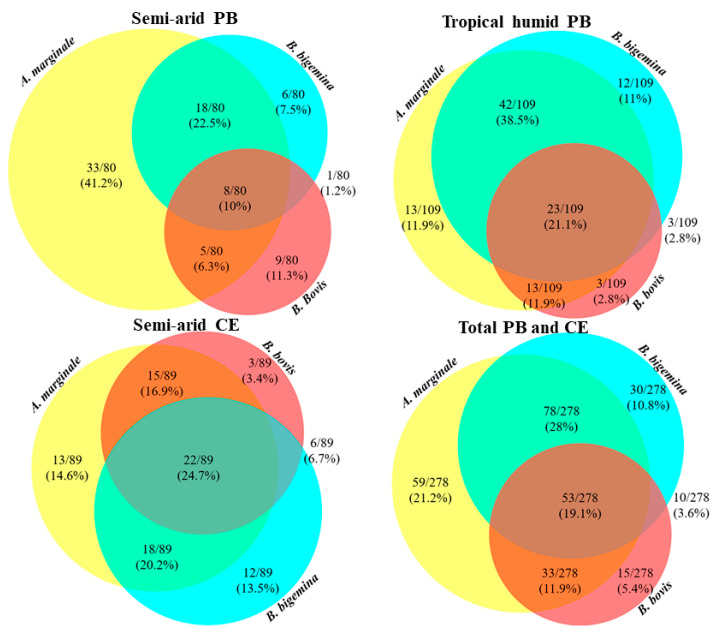
Venn diagrams of infections and coinfections by *A. marginale*, *B. bovis*, and *B. bigemina* in cattle from all three regions and the sum of the three regions. Absolute and percentage values represent animals positive for one, two, or three simultaneous infections.

The analysis of coinfections revealed that, among the animals positive for some CTF agents, the simultaneous occurrence of *B. bovis* + *B. bigemina* was the lowest coinfection in all regions, with emphasis on the semi-arid region of Paraíba, which obtained only 1.3% (1/80), while the tropical humid region of Paraíba presented 2.8% (3/109) and the semi-arid region of Ceará obtained 6.7% (6/89). In total, this combination was detected in 3% of the animals. It was also observed that *A. marginale* + *B. bigemina* was the most common coinfection overall, with 28.0% (78/278) in all three regions, while being surpassed only by triple infection in the semi-arid region of Ceará, in which *A. marginale* + *B. bigemina* represented 20.2% (18/89) and triple infection represented 24.7% (22/89) (Figure 2).

**Figure 3:**
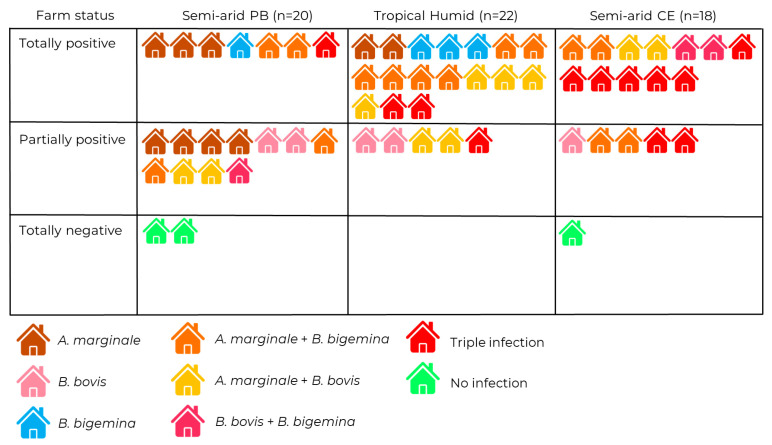
Farm status (totally positive, partially positive, or totally negative) for infections and/or co-infections by *A. marginale*, *B. bovis*, and/or *B. bigemina* in cattle in the semi-arid regions of Paraíba and Ceará and tropical humid region of Paraíba.

The diversity of infection profiles of each farm was observed, ranging from single infections by *A. marginale*, *B. bovis*, or *B. bigemina*, to double combinations and even simultaneous triple infection by all three agents (Figure 3). The semi-arid region of Paraíba had the fewest entirely positive properties (7/20) and the highest number of entirely negative properties (2/20). In contrast, the tropical humid region had the highest number of entirely positive properties (17/21), with no properties entirely free of CTF agents.

It was observed that in the semi-arid region of Ceará, only one property presented singular infections of only one of the CTF agents (1/18). Similarly, this region presented the largest number of properties with all three CTF agents, with 44.4% (8/18) of the properties containing infections for all agents.

Regional variations were observed between municipalities within each region. In the semi-arid region of Paraíba, municipalities such as Bom Sucesso and São Francisco presented low detection rates (≤33.3%), with a complete absence of *B. bovis* or *B. bigemina* in some cases. In contrast, locations such as Marizópolis and Cachoeira dos Índios presented 100.0% positive samples. In the Ceará semi-arid region, Icó stood out for the high frequency of properties with all three agents, while Barro had a lower proportion of positive samples (Table 3). In the tropical humid region, municipalities such as João Pessoa and Remígio showed prevalence rates close to 100.0%, with no infection-free properties.

Univariate analysis revealed significant associations between several epidemiological variables and the occurrence of CTF (Table 4). Sharing pastures (*p* = 0.0029), the presence of horn flies (*p* = 0.0015), and the purchase and introduction of animals without quarantine (*p* = 0.0042) were identified as risk factors at the regional level. Particularly in the semi-arid region of Paraíba, the reuse of needles (*p* = 0.026) and the sharing of pastures (*p* = 0.007) were significantly associated with infection.

Multivariate analysis identified that needle reuse increased the risk of infection by 5.8 times (CI 2.62–13.90; 95% CI); sharing pastures tripled the risk (OR = 3.21; CI 1.08–11.25; 95% CI) and the presence of horn flies presented the highest relative risk (OR = 7.23; CI 3.05–18.86; 95% CI). Furthermore, purchasing animals increased the risk by 5.4 times (CI 2.17–14.93; 95% CI) (Table 5).

## 4. Discussion

### 4.1. Regional Prevalence Patterns and Environmental Context

The high positivity for at least one agent of the CTF complex (82.7% [278/336]) demonstrates the wide molecular circulation of *Anaplasma marginale*, *Babesia bovis,* and *B. bigemina* in the studied regions. Among the detected pathogens, *A. marginale* showed the highest prevalence, consistent with its broad range of transmission routes [13]. This also corroborates with other studies performed in the semi-arid region of Paraíba [11,12]; although these studies used blood smears for diagnosis instead of PCR, the most common pathogen found was *A. marginale*. This bacterium is capable of producing significant hematologic and economic impacts, including anemia, weight loss, reduced milk yield, and mortality in susceptible cattle [16].

In addition to the biological transmission by *R. microplus*, *A. marginale* can also be spread mechanically by biting flies, such as *Haematobia irritans* and iatrogenically through the reuse of contaminated needles and instruments [13]. These alternative routes of dissemination may facilitate the maintenance of asymptomatic carrier animals within herds, which act as persistent sources of infection even in areas with lower tick challenge.

The positivity rates for *B. bovis* and *B. bigemina* varied according to the region. The semi-arid region of Paraíba displayed almost half the amount of positive animals obtained in the semi-arid region of Ceará (Table 2). Although both regions are characterized by a semi-arid climate and are geographically close to each other, there is a difference in rainfall throughout the year. Ceará has more dispersed rainfall throughout the year and higher relative humidity [19], while in the semi-arid region of Paraíba, annual rainfall is concentrated mainly between February and May, in addition to high evapotranspiration rates that reduce relative humidity [18,20]. These climatic differences and more uniform distribution of rainfall may explain the greater dissemination of *Babesia* spp. in the Ceará region, as it creates favorable conditions for year-round tick development, and consequently, of *B. bovis* and *B. bigemina* [26,27].

A previous study in the semi-arid region of Paraíba reported that only 61.0%, 17.1%, and 7.3% of the calves became infected by *A. marginale, B. bigemina,* and *B. bovis* during their first year of life [28], reflecting the overall unfavorable conditions for year-long transmission of CTF agents by ticks or flies in the region [2,28].

Distinct patterns of infections were observed among CTF agents (Figure 2). *Anaplasma marginale* had the highest prevalence as a single infection (17.5%; 59/336), followed by *B. bigemina* (8.9%; 30/336), while *B. bovis* alone was the least prevalent (4.4%; 15/336). This distribution may reflect differences in transmission mechanisms, with *A. marginale* being capable of spreading by both biological and mechanical vectors [13], while *Babesia* spp. species depend strictly on the tick *R. microplus* for their dissemination [29].

### 4.2. Mixed Infections and Epidemiological Dynamics

Triple coinfection (*B. bovis* + *B. bigemina* + *A. marginale*) was detected in 15.8% (53/336) of the animals. Interestingly, the association between *B. bigemina* and *A. marginale* (23.2%; 78/336) was significantly more common than between the two *Babesia* spp. species (3.0%; 10/336). This difference could be related to the higher overall prevalence of *A. marginale* (as discussed above) and due to the generally higher levels of parasitemia of *B. bigemina* than *B. bovis* in cattle. This is reflected in the sensitivity of direct diagnostic assays targeting these two agents [30,31]. It could also be related to the fact that previous studies have reported *B. bigemina* infection rates higher than those of *B. bovis* among unfed larvae of *R. microplus*, which would result in higher inoculation rates of *B. bigemina* in animals, resulting in a higher incidence of this species when compared to *B. bovis* [32,33,34].

The epidemiological overlap between the transmission routes of *Babesia* spp. and *A. marginale* may also explain the relatively lower frequency of double infections involving *B. bovis* and *B. bigemina* when compared to triple infections. In endemic environments where *R. microplus* is abundant, the transmission of *B. bovis* and *B. bigemina* occurs as readily as that of *A. marginale*, since the tick is capable of transmitting all three pathogens [4]. Moreover, because *A. marginale* can be transmitted not only biologically by *R. microplus* but also mechanically by biting flies and contaminated instruments, it easily integrates into existing *Babesia* spp. transmission cycles [16,17]. It is therefore plausible that situations in which both *Babesia* species are present without *A. marginale* occur less frequently, given the multiple routes available for *A. marginale* dissemination. Furthermore, primary infection with *A. marginale* can be lifelong in cattle [35], in contrast to the limited infection (up to months or a few years) with *B. bigemina* and *B. bovis* in the absence of reinfection episodes [36].

In addition to the harm that multiple infections cause to animals, they also pose practical challenges to diagnosis and treatment. Because the clinical signs caused by the agents of the CTF complex are quite similar, it is difficult to distinguish which agent is involved solely through clinical observation [8,14]. Furthermore, the low sensitivity of the most accessible diagnostic methods makes it difficult to choose the most appropriate treatment, leading to treatments being administered based on the presence of all agents in the complex, often with a combination of babesicides and anaplasmicides, without confirming the specific agent [16,37].

### 4.3. Therapeutic Context and Acaricide/Drug Resistance

As treatment involves a complex decision on which medication to use, the issue of indiscriminate treatments and drug resistance emerges as a critical concern in the epidemiology of the CTF complex [16]. Several studies have reported high levels of resistance to multiple insecticides in field populations of *H. irritans* [38,39] and *R. microplus* [2,5,26,27,40]. Such resistance undermines chemical control programs and allows horn flies to remain active vectors of *A. marginale* even in treated herds [38]. Likewise, recent investigations have shown that therapeutic use of tetracyclines, including oxytetracycline and chlortetracycline at approved doses, often fails to completely eliminate persistent *A. marginale* infections [41].

### 4.4. Microgeographic Patterns of Infection Distribution

In this study, a farm in the municipality of Vieirópolis had recurrent clinical CTF in its herd and was frequently treated with both anaplasmicides and babesicides. As a result, the farm was free of *Babesia* spp. but suffered from drug-resistant *A. marginale*. These findings indicate that both indiscriminate acaricide application and empirical antibiotic treatments may contribute to the maintenance of chronically infected animals and the continuous circulation of pathogens within herds.

In the semi-arid region of Paraíba, municipalities with lower prevalence rates, such as Bom Sucesso and Catolé do Rocha (Table 3), probably reflect areas where seasonal rainfall and low relative humidity prevent or reduce the efficiency of the tick reproductive cycle, reducing the continuous exposure of livestock [27].

In contrast, Icó (CE) and other municipalities in Ceará, despite having a semi-arid climate, recorded multiple infections in virtually all tested animals. The municipality of Bernardino Batista, which borders the states of Paraíba and Ceará and borders Icó (CE), also had 100% of its animals infected with CTF agents. Regional proximity can influence outbreaks and allow the migration of agents, such as through the regional buying and selling of animals. Similarly, neighboring municipalities like Santa Cruz (PB), São Francisco (PB), and Bom Sucesso (PB) had prevalence rates below 50.0%. This indicates that there are geographic foci of high or low positivity in the regions studied.

In the tropical humid region, the universal positivity in João Pessoa (PB), Remígio (PB) and the high prevalence in Esperança (PB) indicate consolidated enzootic stability. In regions of high humidity, transmission occurs year-round, ensuring early exposure of calves and reducing the occurrence of acute clinical outbreaks, but maintaining a high potential for chronic infections and coinfections [5]. This stability can, paradoxically, protect against severe outbreaks while perpetuating subclinical losses and contributing to the subclinical spread of agents [42].

In municipalities with 100% positivity, such as Icó and João Pessoa, where virtually all animals are infected, coinfections become the norm, not the exception. This overlap of pathogens, even in contexts of enzootic stability, can influence the severity of clinical conditions when there are immune failures or stressful conditions [43].

### 4.5. Management Practices and Farm-Level Risk Factors

The presence of horn flies (*H. irritans*) emerged as the main risk factor (OR = 7.23), which can be explained by their well-established role in the mechanical transmission of *A. marginale* [13,42,44]. These flies feed repeatedly on multiple animals, often hundreds of times per day, producing small skin wounds that facilitate the transfer of infected erythrocytes between hosts [44]. Their high population turnover, reaching up to 30 generations per year in the semi-arid region [45], enables continuous pathogen circulation throughout the year.

In the Paraíba semi-arid region, pasture sharing (OR = 3.21) was particularly relevant, as this practice can lead animals to graze in areas potentially infested with ticks, effectively importing these parasites from neighboring properties. This finding contrasts with the tropical humid region, where no single variable showed significance, suggesting that environmental factors such as constant humidity may equalize infection risks [27].

It was observed that the reuse of needles was the second most important risk factor (OR = 5.81), corroborating previous studies that identified this practice as the main route of iatrogenic transmission of *A. marginale* [46]. This is a critical and often overlooked biosecurity failure in daily farm operations. Needle reuse can be attributed to a combination of factors, including economic constraints, lack of awareness among producers about the associated risks, and the persistent use of metal applicators designed for reusable needles, which inadvertently encourage this hazardous practice [47]. Beyond the transmission of *A. marginale*, the reuse of needles poses a substantial risk for disseminating other blood-borne pathogens within and between herds, effectively turning routine veterinary procedures into potential disease-spreading events [46].

The results revealed that beef production systems had a lower risk of CTF (OR = 0.28) compared to dairy systems. This difference can be attributed to the shorter production cycle and the dynamics of infection, in which beef cattle are typically slaughtered younger (24–36 months), limiting the time of exposure to pathogens. In contrast, dairy cows remain in the herd for several years, accumulating chronic infections [48,49].

The dynamics of infection indicate that beef animals develop temporary immunity after acute infections, while dairy cows, constantly under metabolic stress from lactation and the continuous production chain, make these animals more susceptible to reinfections/recrudescence and clinical manifestations [50].

Animal management and ecological factors are critical determinants of CTF transmission dynamics. Practices such as needle reuse, inadequate quarantine of newly purchased animals, pasture sharing between farms, and the presence of mechanical vectors have consistently been associated with higher infection risk and the maintenance of chronically infected animals. Needle reuse can be mitigated by adopting single-use syringes and applying aseptic procedures during vaccination, medication, and routine handling to prevent iatrogenic transmission of *A. marginale* [35].

The introduction of new animals represents another relevant risk factor. Establishing a 30-day quarantine period, combined with clinical and laboratorial screening before herd integration, helps reduce the entry of infected carriers [35]. Pasture sharing and unrestricted movement of cattle between properties facilitate the spread of ticks and mechanical vectors; therefore, producers should adopt controlled grazing schemes and rotational practices that disrupt tick life cycles [51,52].

In highly infested areas, strategic burning of contaminated paddocks, when properly regulated and safely executed, can lower the density of *R. microplus* larvae [52]. Additionally, integrated fly management programs targeting *H. irritans,* including manure management, strategic insecticide rotation, and traps, may reduce mechanical transmission pressure [52,53]. Altogether, combining targeted vector control with pasture and environmental management can reduce pathogen circulation and lessen the clinical and economic impact of CTF.

## 5. Conclusions

Cattle tick fever remains a significant challenge for cattle farming in the Northeast region of Brazil, with a high prevalence in all regions analyzed. *Anaplasma marginale* was the most frequent agent, while *Babesia* spp. infections were more dependent on environmental conditions favorable to the *R. microplus* tick. The spatial analysis revealed marked regional differences, identifying foci of infection in specific areas within the semi-arid and tropical humid regions. These local foci, often involving neighboring municipalities, suggest that pathogen spread may be facilitated by animal movement and trade between farms within the same epidemiological region. The main risk factors identified were needle reuse, the presence of horn flies, the purchase and introduction of animals without quarantine, and pasture sharing. Furthermore, more than half of the cattle tested positive for multiple agents, and these coinfection patterns hinder both accurate diagnosis and the selection of appropriate treatment.

## Figures and Tables

**Table 1:** Species-specific primer description of each primary and secondary (nested) reaction. Full thermocycler conditions for the reactions of *A. marginale*, *B. bovis*, and *B. bigemina*.

Code	Sequence			Hemoparasite
Primary PCR				
MSP5-F	5′-CGC AGA TCT AGC AAA ATC GGC GAG AGG TTT ACC ACT TC-3′			*A. marginale*
MSP5-R	5′-GCG CTG CAG TGG CGC AAA ATG CCC GAC ATA CC -3′			
BoF	5′-CAC GAG GAA GGA ACT ACC GAT GTT GA-3′			*B. bovis*
BoR	5′-CCA AGG AGC TTC AAC GTA CGA GGT CA-3′			
Bi1A	5′-CAT CTA ATT TCT CTC CAT ACC CCT CC-3′			*B. bigemina*
Bi1B	5′-CCT CGG CTT CAA CTC TGA TGC CAA AG-3′			
Nested PCR				
BoFN	5′-TCA ACA AGG TAC TCT ATA TGG CTA CC-3′			*B. bovis*
BoRN	5′-CTA CCG AGC AGA ACC TTC TTC ACC AT-3′			
Bi1AN	5′-CGC AAG CCC AGC ACG CCC CGG TGC-3′			*B. bigemina*
Bi1BN	5′-CCG ACC TGG ATA GGC TGT GTG ATG-3′			
Thermocycler conditions				
Initial denaturation	Denaturation	Annealing	Extension	Final extension
95 °C for 1 min	95 °C for 15 s	55 °C for 15 s	72 °C for 30 s	72 °C for 4 min
1×	35×	35×	35×	1 time

**Table 2:** Prevalence of the cattle tick fever (CTF) agents, *B. bovis*, *B. bigemina,* and *A. marginale,* in cattle from three regions of Northeast Brazil (semi-arid regions of Paraíba and Ceará and tropical humid region of Paraíba).

Parasite	Semi-Arid PB Positive/Total (%)	Tropical Humid PB Positive/Total (%)	Semi-Arid CE Positive/Total (%)	Total (%)
*B. bovis*	9/120 (7.5%)	3/115 (2.6%)	3/101 (3.0%)	15/336 (4.4%)
*B. bigemina*	6/120 (5.0%)	12/115 (10.5%)	12/101 (11.9%)	30/336 (8.9%)
*A. marginale*	33/120 (27.5%)	13/115 (11.3%)	13/101 (12.9%)	59/336 (17.5%)
*B. bovis* + *B. bigemina*	1/120 (0.8%)	3/115 (2.6%)	6/101 (5.9%)	10/336 (3.0%)
*B. bovis* + *A. marginale*	5/120 (4.2%)	13/115 (11.3%)	15/101 (14.8%)	33/336 (9.8%)
*B. bigemina* + *A. marginale*	18/120 (15%)	42/115 (36.5%)	18/101 (17.8%)	78/336 (23.2%)
Triple infection	8/120 (6.6%)	23/115 (20.0%)	22/101 (21.8%)	53/336 (15.8%)
CTF *	80/120 (66.6%)	109/115 (94.8%)	89/101 (88.1%)	278/336 (82.7%)

*: animals positive for at least one of the three CTF agents.

**Table 3:** Municipal proportion of collections and infections by *A. marginale*, *B. bovis,* and *B. bigemina* in cattle from the semi-arid (PB and CE) and tropical humid (PB) regions.

Semi-Arid Region PB	Farms	Total Animals	Samples Collected (%)	*A. marginale* (%)	*B. bovis* (%)	*B. bigemina* (%)	Total (%)
Bernardino Batista	3	67	26.8 (18/67)	72.2 (13/18)	33.3 (6/18)	55.5 (10/18)	100.0 (18/18)
Bom Sucesso	1	83	6.9 (6/83)	16.6 (1/6)	0.0 (0/6)	0.0 (0/6)	16.6 (1/6)
Cachoeira dos Índios	1	100	6.0 (6/100)	66.6 (4/6)	50.0 (3/6)	50.0 (3/6)	100.0 (6/6)
Catolé do Rocha	5	365	8.2 (30/365)	43.3 (13/30)	10.0 (3/30)	0.0 (0/30)	43.3 (13/30)
Coremas	1	21	28.5 (6/21)	50.0 (3/6)	33.3 (2/6)	66.6 (4/6)	100.0 (6/6)
Marizópolis	1	110	5.4 (6/110)	100.0 (6/6)	16.6 (1/6)	50.0 (3/6)	100.0 (6/6)
Patos	2	89	13.5 (12/89)	41.6 (5/12)	25.0 (4/12)	33.3 (4/12)	66.6 (8/12)
Santa Cruz	1	25	24.0 (6/25)	33.3 (2/6)	0.0 (0/6)	50.0 (3/6)	50.0 (3/6)
Sousa	2	125	9.6 (12/125)	66.6 (8/12)	8.3 (1/12)	25.0 (3/12)	66.6 (8/12)
São Francisco	1	27	22.2 (6/27)	0.0 (0/6)	33.3 (2/6)	0.0 (0/6)	33.3 (2/6)
S.J. Espinharas	1	650	0.9 (6/650)	66.6 (4/6)	16.6 (1/6)	50.0 (3/6)	66.6 (4/6)
Vieirópolis	1	200	3.0 (6/200)	83.3 (5/6)	0.0 (0/6)	0.0 (0/6)	83.3 (5/6)
Total Semi-arid PB	20	1862	6.4 (120/1862)	53.3 (64/120)	19.2 (23/120)	27.5 (33/120)	66.6 (80/120)
Tropical Humid region PB							
Areia	5	151	17.2 (26/151)	69.2 (18/26)	26.9 (7/26)	53.8 (14/26)	82.1 (23/28)
Esperança	3	112	15.1 (17/112)	82.3 (14/17)	52.9 (9/17)	70.5 (12/17)	94.1 (16/17)
Fagundes	3	138	11.6 (16/138)	75.0 (12/16)	25.0 (4/16)	62.5 (10/16)	100.0 (16/16)
João Pessoa	5	133	19.5 (26/133)	88.4 (23/26)	34.6 (9/26)	76.9 (20/26)	100.0 (26/26)
Remígio	6	151	19.8 (30/151)	80.0 (24/30)	43.3 (13/30)	80.0 (24/30)	93.3 (28/30)
Total Tropical Humid PB	22	685	16.8 (115/685)	79.1 (91/115)	36.5 (42/115)	69.5 (80/115)	94.8 (109/115)
Semi-arid region CE							
Baixio	3	114	14.1 (16/114)	75.0 (12/16)	50.0 (8/16)	43.75 (7/16)	87.5 (14/16)
Barro	3	123	13.8 (17/123)	41.1 (7/17)	0.0 (0/17)	35.3 (6/17)	58.8 (10/17)
Icó	7	903	4.4 (40/903)	87.5 (35/40)	52.5 (21/40)	75.0 (30/40)	100.0 (40/40)
Iguatu	1	38	15.8 (6/38)	0.0 (0/6)	83.3 (5/6)	50.0 (3/6)	83.3 (5/6)
Juazeiro do Norte	1	49	12.2 (6/49)	50.0 (3/6)	33.3 (2/6)	83.3 (5/6)	83.3 (5/6)
L. da Mangabeira	1	10	50.0 (5/10)	60.0 (3/5)	20.0 (1/5)	40.0 (2/5)	100.0 (5/5)
Orós	1	20	25.0 (5/20)	60.0 (3/5)	80.0 (4/5)	80.0 (4/5)	100.0 (5/5)
Santana do Cariri	1	150	4.0 (6/150)	83.3 (5/6)	83.3 (5/6)	16.6 (1/6)	83.3 (5/6)
Total Semi-arid CE	18	1407	7.2 (101/1407)	67.3 (68/101)	45.5 (46/101)	57.4 (58/101)	88.1 (89/101)
Total	60	3954	8.5 (336/3954)	66.4 (223/336)	33.0 (111/336)	50.5 (170/336)	82.7 (278/336)

**Table 4:** Univariate analysis of risk factors for CTF in cattle in the semi-arid regions of Paraíba and Ceará and tropical humid region of Paraíba and the sum of the regions studied.

Variable	Category	Semi-Arid PB (*p*-Value)	Tropical Humid PB (*p*-Value)	Semi-Arid CE (*p*-Value)	Region (*p*-Value)
Age	0–2 years old	0.98	0.57	0.3	0.3
	2–5 years old				
	5+ years old				
Gender	Male/female	0.99	0.11	0.99	0.99
Breed	Zebu/Mixed	<0.001 *	<0.001 *	0.008 *	0.03 *
Type of exploration	Beef/Dairy	0.004 *	0.19	0.99	<0.001 *
Production system	Intensive/Extensive	0.38	0.99	0.99	<0.041 *
CTF History	Yes/No	0.63	0.40	0.99	0.25
Reuses needles	Yes/No	0.026 *	0.99	0.50	0.024 *
Share pasture	Yes/No	0.007 *	0.66	0.35	0.003 *
Horn flies	Present/Absent	0.23	0.99	<0.01 *	0.002 *
Animal purchase	Yes/No	0.14	0.18	0.99	0.004 *
Bull rotation between farms	Yes/No	0.34	0.19	0.07	0.023 *

*: Statistically significant.

**Table 5:** Multivariate analysis of risk factors for CTF in cattle in the semi-arid regions of Paraíba and Ceará and tropical humid region of Paraíba.

Variable	Odds Ratio (95% CI)	*p*-Value	Significance
Intercept	0.26 (0.06–1.04)	0.063	ns
Type of exploration	0.28 (0.15–0.52)	<0.001	*
Reuses needles	5.81 (2.62–13.90)	<0.001	*
Share pasture	3.21 (1.08–11.25)	0.048	*
Horn flies	7.23 (3.05–18.86)	<0.001	*
Animal purchase	5.37 (2.17–14.93)	<0.001	*

ns: not significant; *: statistically significant.

## Data Availability

The data presented in this study are available from the corresponding author upon reasonable request. No publicly archived datasets were created or analyzed in this study.
